# iTRAQ-based quantitative proteomic analysis of the antibacterial mechanism of silver nanoparticles against multidrug-resistant *Streptococcus suis*

**DOI:** 10.3389/fmicb.2023.1293363

**Published:** 2023-11-15

**Authors:** Baoling Liu, Dingyu Liu, Tianbao Chen, Xiaohu Wang, Hua Xiang, Gang Wang, Rujian Cai

**Affiliations:** ^1^Institute of Animal Health, Guangdong Academy of Agricultural Sciences, Key Laboratory of Livestock Disease Prevention of Guangdong Province, Scientific Observation and Experiment Station of Veterinary Drugs and Diagnostic Techniques of Guangdong Province, Ministry of Agriculture and Rural Affairs, Guangzhou, China; ^2^College of Animal Science and Technology, Zhongkai University of Agriculture and Engineering, Guangzhou, China

**Keywords:** AgNPs, iTRAQ, *Streptococcus suis*, antibacterial mechanism, Biofilm

## Abstract

**Background:**

The increase in antibiotic resistance of bacteria has become a major concern in clinical treatment. Silver nanoparticles (AgNPs) have significant antibacterial effects against *Streptococcus suis*. Therefore, this study aimed to investigate the antibacterial activity and mechanism of action of AgNPs against multidrug-resistant *S. suis*.

**Methods:**

The effect of AgNPs on the morphology of multidrug-resistant *S. suis* was observed using scanning electron microscopy (SEM). Differentially expressed proteins were analyzed by iTRAQ quantitative proteomics, and the production of reactive oxygen species (ROS) was assayed by H_2_DCF-DA staining.

**Results:**

SEM showed that AgNPs disrupted the normal morphology of multidrug-resistant *S. suis* and the integrity of the biofilm structure. Quantitative proteomic analysis revealed that a large number of cell wall synthesis-related proteins, such as penicillin-binding protein and some cell cycle proteins, such as the cell division protein FtsZ and chromosomal replication initiator protein DnaA, were downregulated after treatment with 25 μg/mL AgNPs. Significant changes were also observed in the expression of the antioxidant enzymes glutathione reductase, alkyl hydroperoxides-like protein, α/β superfamily hydrolases/acyltransferases, and glutathione disulfide reductases. ROS production in *S. suis* positively correlated with AgNP concentration.

**Conclusion:**

The potential antibacterial mechanism of AgNPs may involve disrupting the normal morphology of bacteria by inhibiting the synthesis of cell wall peptidoglycans and inhibiting the growth of bacteria by inhibiting the cell division protein FtsZ and Chromosomal replication initiator protein DnaA. High oxidative stress may be a significant cause of bacterial death. The potential mechanism by which AgNPs inhibit *S. suis* biofilm formation may involve affecting bacterial adhesion and interfering with the quorum sensing system.

## 1. Introduction

*Streptococcus suis* is an important zoonotic pathogen with worldwide prevalence and is considered to be one of the most important bacterial pathogens causing significant economic losses in the swine industry (Segura, 2020). As with most pathogens, the ability of *S. suis* to form biofilms plays a significant role in its virulence and drug resistance (Wang et al., 2018). Currently, the treatment of *S. suis* infections relies on antibiotics; however, drug resistance is a concern. Data suggest that available veterinary drugs, such as ampicillin, cefepime, cefotaxime, ceftiofur, ceftriaxone, chloramphenicol, florfenicol, gentamicin, penicillin, and tiamulin, tend to be less effective in treating *S. suis* infections (Lunha et al., 2022). *S. suis* has an extremely high rate of resistance to tetracyclines, lincosamides, and macrolides, and resistance has spread globally (Uruen et al., 2022). Therefore, there is an urgent need to develop efficient alternatives to antibiotics.

Silver has strong antimicrobial potential and has been used since ancient times (Rai et al., 2012). AgNPs are now considered a viable alternative to antibiotics and appear to have appreciable potential to address the concern of bacterial multidrug resistance (Franci et al., 2015). AgNPs show distinct antibacterial and anti-biofilm formation effects on bacteria. For example, it has been proven that AgNPs could play antimicrobial roles in the multidrug-resistant (MDR) *Pseudomonas aeruginosa* and the main mechanism involves the disequilibrium of oxidation and antioxidation processes and the failure to eliminate the excessive ROS (Liao et al., 2019). Siddique et al. provided evidence of AgNPs being safe antibacterial and antibiofilm compounds against MDR *Klebsiella pneumoniae* (Siddique et al., 2020). Farouk et al. demonstrated the effective ability of AgNPs to fight MDR *Salmonella* spp. *in vitro* and *in vivo* without adverse effects (Farouk et al., 2020). AgNPs have antibacterial activity against multidrug-resistant bacteria pathogens, such as *Vibrio cholerae*, *Staphylococcus aureus, Streptococcus pyogenes*, *Escherichia coli*, and *Klebsiella pneumoniae* (Chinnathambi et al., 2023). The mechanism of antimicrobial activity of AgNPs involves four steps: (i) adhesion of AgNPs to cell wall/membrane and their disruption; (ii) intracellular penetration and damage; (iii) oxidative stress; and (iv) modulation of signal transduction pathways (Wahab et al., 2021; Tripathi and Goshisht, 2022). In our previous study, AgNPs showed significant activity against *S. suis in vitro* (Liu et al., 2022), however, the mechanism of AgNPs against *S. suis* remains unclear.

The occurrence of disease and the therapeutic effect of drugs are always accompanied by fluctuations and changes in numerous proteins. The application of quantitative proteomics can enable visualization of the up-regulation and down-regulation of differential proteins through charts, and provide functional annotations to intuitively analyze the possible mechanism of action of drugs (Saleh et al., 2019). In particular, iTRAQ technology has good applicability in the study of antibacterial mechanisms.

In this study, ultrastructural observations using scanning electron microscopy (SEM), fluorescence microscopy, and iTRAQ-based quantitative proteomics were used to investigate the antibacterial mechanism of action of AgNPs against multidrug-resistant *S. suis*. The findings can provide significant insights into the molecular mechanism of AgNPs against *S.suis.*

## 2. Materials and methods

### 2.1. AgNPs and culture conditions of *Streptococcus suis*

The ready-to-use AgNP stock solution (concentration: 30000 μg/mL, size: 5–7 nm, density: 1.01 g/cm^3^) was provided by Guangdong Shunde Zhengshanchuan Biotechnology Co., Ltd. (China).

A *S. suis* type 2 strain isolated from a diseased pig was employed and preserved in our laboratory. In our previous study, we identified it as an MDR bacterium that exhibits resistance to various antibiotics, such as tetracycline, doxycycline, penicillin, florfenicol, cefotaxime, kanamycin, and lincomycin, and that the minimum inhibitory concentration (MIC) of AgNPs against it is 25 μg/mL (Liu et al., 2022). *S. suis* was cultured in trypticase soy broth (TSB) or maintained on trypticase soy agar (TSA) supplemented with 5% bovine serum (Gibco, Auckland, New Zealand) at 37°C.

### 2.2. AgNP treatment and preparations for SEM observation

To observe the impact of AgNPs on the morphology of *S. suis*, *S. suis* bacteria were proliferated to 1 × 10^8^ CFU/mL. After being exposed to 6.25, 12.5, 25, 50, and 100 μg/mL AgNPs for 12 h, the culture was precipitated by centrifugation and washed with PBS and then centrifuged; the precipitates were fixed in 2.5% glutaraldehyde for 2 h at 4. Subsequently, the precipitates were centrifuged, and washed with sterile PBS. The precipitates were then sequentially dehydrated through a series of alcohols (30, 50, 70, 90, and 100%) to the critical point. After coating with gold, the samples were examined using SEM (Zeiss Sigma 300).

To observe the effect of AgNPs on the biofilms of *S. suis*, 1 cm × 1 cm coverslips were ultrasonically cleaned for 1 h, and surface impurities and grease were removed and sterilized with high-pressure steam. A 6-well plate was prepared by placing a sterile coverslip in each hole, and then adding 1 mL of bacterial solution to cover the surface of the coverslips. The culture plate was cultured in an incubator at 37°C for 24 h, and the supernatant was discarded. The experimental group was added with TSB containing 25 μg/mL AgNPs and the control group was added with blank TSB. The culture plate was again cultured in an incubator at 37°C for 24 h and the supernatant was discarded. The plates were immersed in 2.5% glutaraldehyde for 3 h, then dehydrated with a series of concentrations of ethanol (30, 50, 70, 90, and 100%), dried, sprayed with gold, and observed with a scanning electron microscope.

### 2.3. iTRAQ quantitative proteome analysis

#### 2.3.1. Protein extraction and iTRAQ labeling

The bacterial cells were treated with 25 μg/mL AgNPs and harvested by centrifugation. Proteins were extracted as follows:

The appropriate amount of sample was weighed and transferred into a 2 mL centrifuge tube, two steel beads and 1XCocktail with an appropriate amount of SDS L3 and EDTA were added, placed on ice for 5 min, and DTT was added at a final concentration of 10 mM. A grinder (frequency 60 HZ, duration 2 min) was used to crush the tissue, which was then centrifuged at 25,000 g*4°C for 15 min, and the supernatant was collected. DTT was added at a final concentration of 10 mM, and the mixture was placed in a water bath at 56°C for 1 h. IAM was added at a final concentration of 55 mM and the mixture was placed in a dark room for 45 min. Cold acetone was added to the protein solution at a ratio of 1:5, placed in a refrigerator at −20°C for 30 min, centrifuged at 25,000 g*4°C for 15 min, and the supernatant was discarded. The precipitate was air-dried, lysis buffer without SDS L3 was added, and a grinder (frequency 60HZ, duration 2 min) was used to promote protein solubilization. This was centrifuged for 15 min at 25,000 g*4°C to collect the supernatant; the supernatant is the protein solution. The proteins were digested, and the resultant peptides were labeled using iTRAQ 8-plex kits (AB Sciex). The untreated samples were labeled as 118, 119, and 121, and the samples treated with AgNPs were labeled as 114, 116, and 118.

#### 2.3.2. LC–MS/MS

The dried peptide samples were reconstituted with mobile phase A (2% ACN, 0.1% FA), centrifuged at 20,000 g for 10 min, and the supernatant was collected for injection. The separation was performed using a Thermo UltiMate 3,000 UHPLC system. The sample was first enriched in a trap column and desalted, and then separated on a self-packed C18 column (75 μm internal diameter, 3 μm column size, 25 cm column length) at a flow rate of 300 nL/min by the following effective gradient: 0 ~ 5 min, 5% mobile phase B (98% ACN, 0.1% FA); 5 ~ 45 min, mobile phase B linearly increased from 5 to 25%; 45 ~ 50 min, mobile phase B increased from 25 to 35%; 50 ~ 52 min, mobile phase B rose from 35 to 80%; 52 ~ 54 min, 80% mobile phase B; 54 ~ 60 min, 5% mobile phase B. The nanolitre liquid-phase separation end was directly connected to a mass spectrometer.

The peptides separated by liquid-phase chromatography were ionized using a Nano ESI source and then passed to a tandem mass spectrometer Q-Exactive HF X (Thermo Fisher Scientific, San Jose, CA) for data-dependent acquisition (DDA) mode detection. The main parameters were set as follows: ion source voltage, 1.9 kV; MS1 scanning range, 350 ~ 1,500 m/z; resolution, 60,000; MS2 starting m/z, 100; and resolution, 15,000. The ion screening conditions for MS2 fragmentation were as follows: charge 2+ to 6+ and the top 20 parent ions with a peak intensity exceeding 10,000. The ion fragmentation mode was HCD and fragment ions were detected using the Orbitrap. The dynamic exclusion time was set to 30 s. The AGC was set to 3E6 in MS1 and 1E5 in MS2.

### 2.4. Detection of ROS

The bacteria of *S.suis* proliferated to 1 × 10^8^ CFU/mL after exposure to 6.25, 12.5, 25, 50, and 100 μg/mL AgNPs for 6 h. ROS was measured by 2′,7′-dichloro fluorescein diacetate (H_2_DCF-DA) based on the method of Liao et al. (2019). Initially, a 10 mM H_2_DCF-DA stock solution in dimethyl sulfoxide was diluted to 1 mM working solution with a TSB medium. The collected bacteria were washed with PBS and suspended in 1.8 mL of PBS. Then the samples were incubated with 200 μL of working solution at 37°C for 30 min in darkness. Subsequently, the cells were harvested, washed, and resuspended in PBS. This bacterial suspension was dropped on a slide and naturally dried in the darkness at room temperature before fluorescence microscopy (ZEISS, Axio vert. A1) detection. The cultured bacteria were lysed using an alkaline lysis buffer and centrifuged at 3,000 rpm for 5 min. Subsequently, 1 mL of lysate supernatant was prepared for fluorescence detection (Multifunctional microplate reader, Thermo Scientific varios) at excitation and emission wavelengths of 470 and 529 nm, respectively.

### 2.5. Statistical analysis

All data are expressed as means ± standard deviation with three biological replicates. GraphPad Prism 8.0 was used to perform one-way ANOVA analysis at *p* ≤ 0.05 and create graphs. Quantification of iTRAQ data was performed using the IQuant software (2.4.0), and the Mascot search engine (v2.3.02, Matrix Sciences, London, United Kingdom) was used to search the UniProt database.

## 3. Results

### 3.1. AgNPs disrupt the morphology and biofilm structure of *Streptococcus suis*

**Figure 1:**
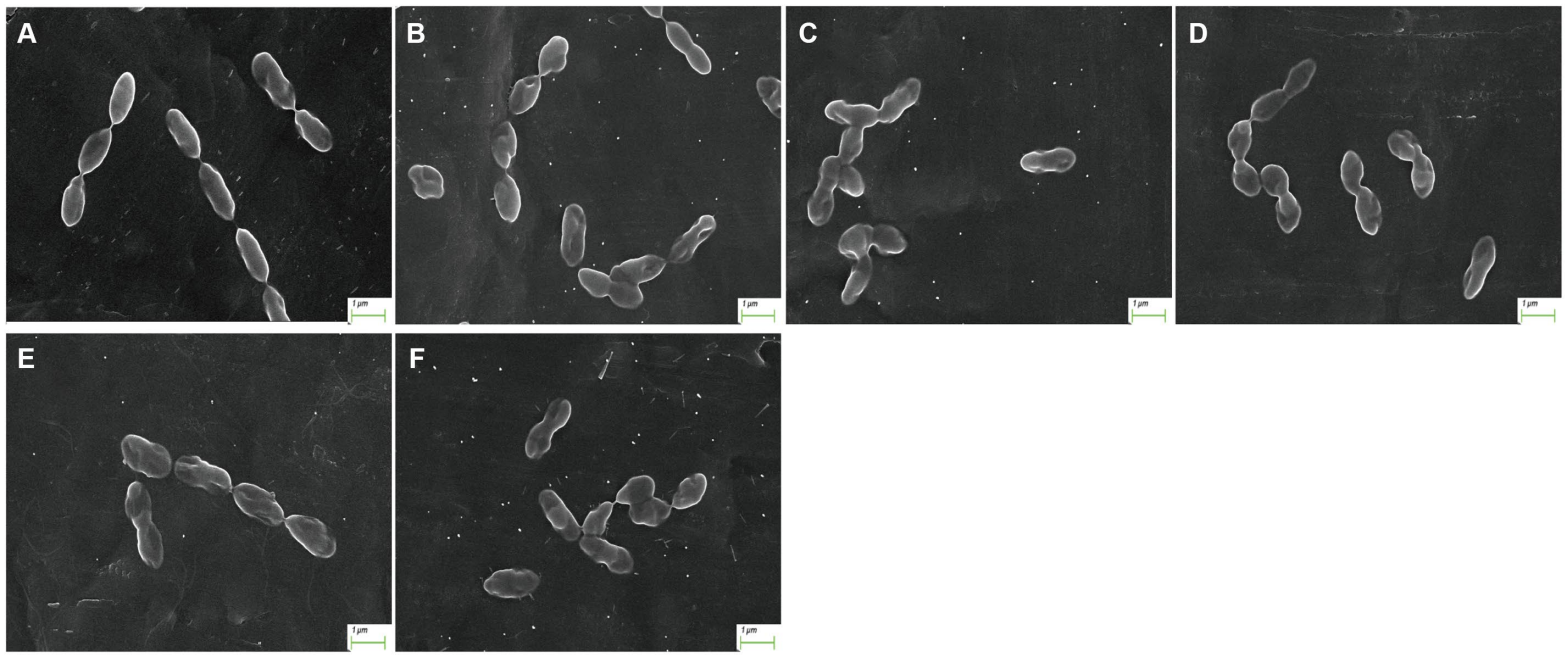
Morphology of *S. suis* after AgNP treatment. **(A)** control, **(B)** 6.25 μg/mL, **(C)** 12.5 μg/mL, **(D)** 25 μg/mL, **(E)** 50 μg/mL, **(F)** 100 μg/mL.

**Figure 2:**
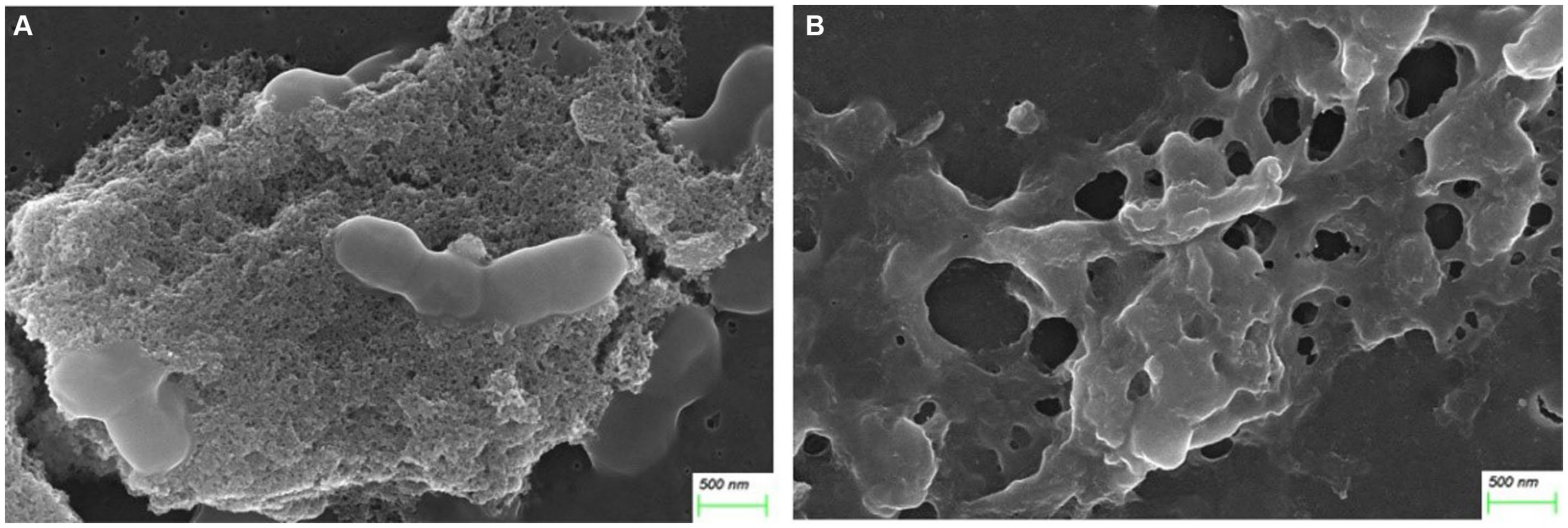
AgNPs disrupted biofilm structure. **(A)** Control. **(B)** Treated with 25 μg/mL AgNPs.

After treatment with AgNP for 12 h, the bacteria of each multidrug-resistant *S. suis* strain were collected for morphological examination using SEM. Compared to the control, pits appeared on the surface of the bacterial cells after AgNP treatment, and the cell morphology was distorted (Figure 1). The analysis showed that AgNPs disrupted the morphology of *S. suis*, and the destruction of cells was aggravated with an increase in AgNP concentration. Further, the structure of the biofilm was destroyed by AgNPs (Figure 2).

### 3.2. Analysis of DEPs after AgNPs treatment

**Figure 3:**
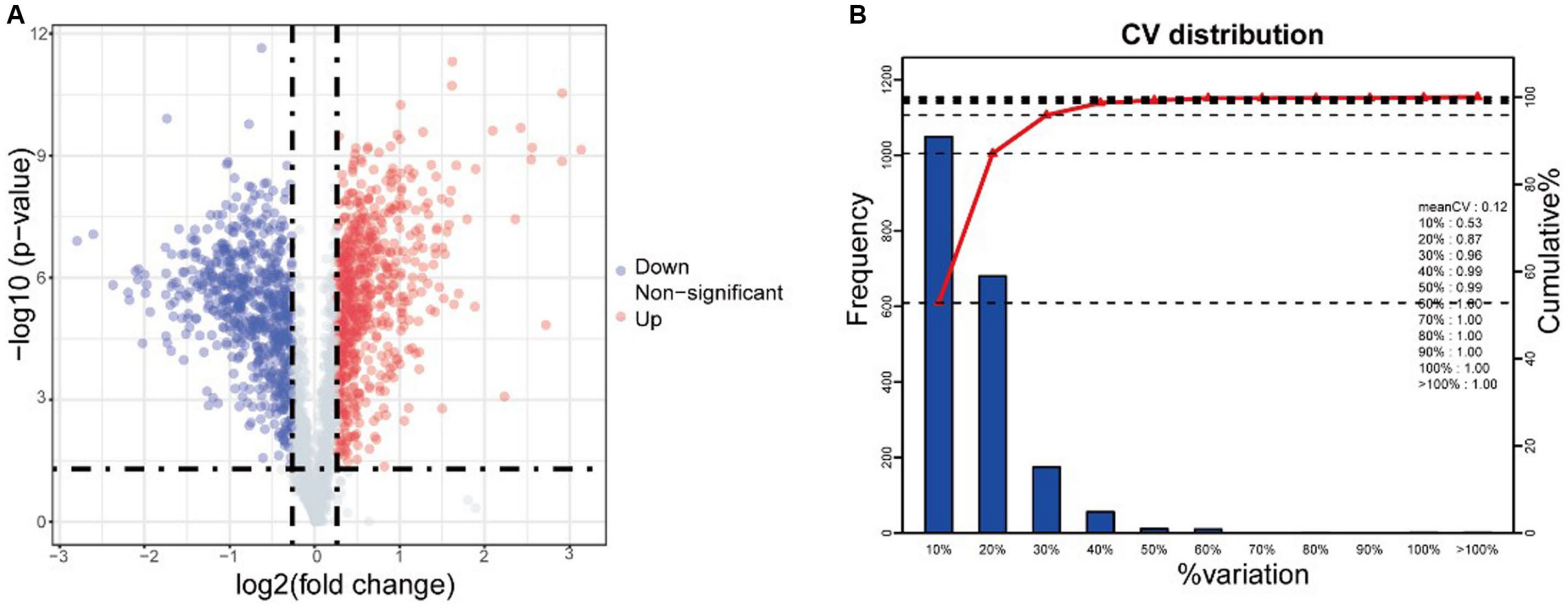
iTRAQ analysis reveals differentially expressed proteins (DEPs) after AgNPs treatment. **(A)** Volcano of differentially expressed proteins, **(B)** CV distribution in replicate. Red and green dots indicate points of interest that display both large- and high-magnitude fold changes, respectively. Red dots indicate significantly upregulated proteins that passed the screening threshold. Blue dots indicate significantly downregulated proteins that passed the screening threshold. Gray dots represent non-significantly differentially expressed proteins.

Quantitative proteomic analysis revealed 1,268 bacterial proteins, and a volcano map of the differentially expressed proteins (DEPs) in *S. suis* was analyzed (X-axis is log2 fold change) (Figure 3A). In total, 633 upregulated and 635 downregulated genes were significantly altered in response to AgNP exposure at 25 μg/mL. This depicts a volcano plot of log2 fold-change (x-axis) versus the -log10 *Q*-value (*y*-axis, representing the probability that the protein is differentially expressed). *Q*-value <0.05 and fold change >1.2 are considered significant differential expression thresholds. Quantitative repeatability was assessed using CV (CV = SD/mean). The lower the CV value, the better the repeatability. The CV value in this experiment was 0.12, indicating good repeatability (Figure 3B).

### 3.3. Functional annotation analysis of DEPs

**Figure 4:**
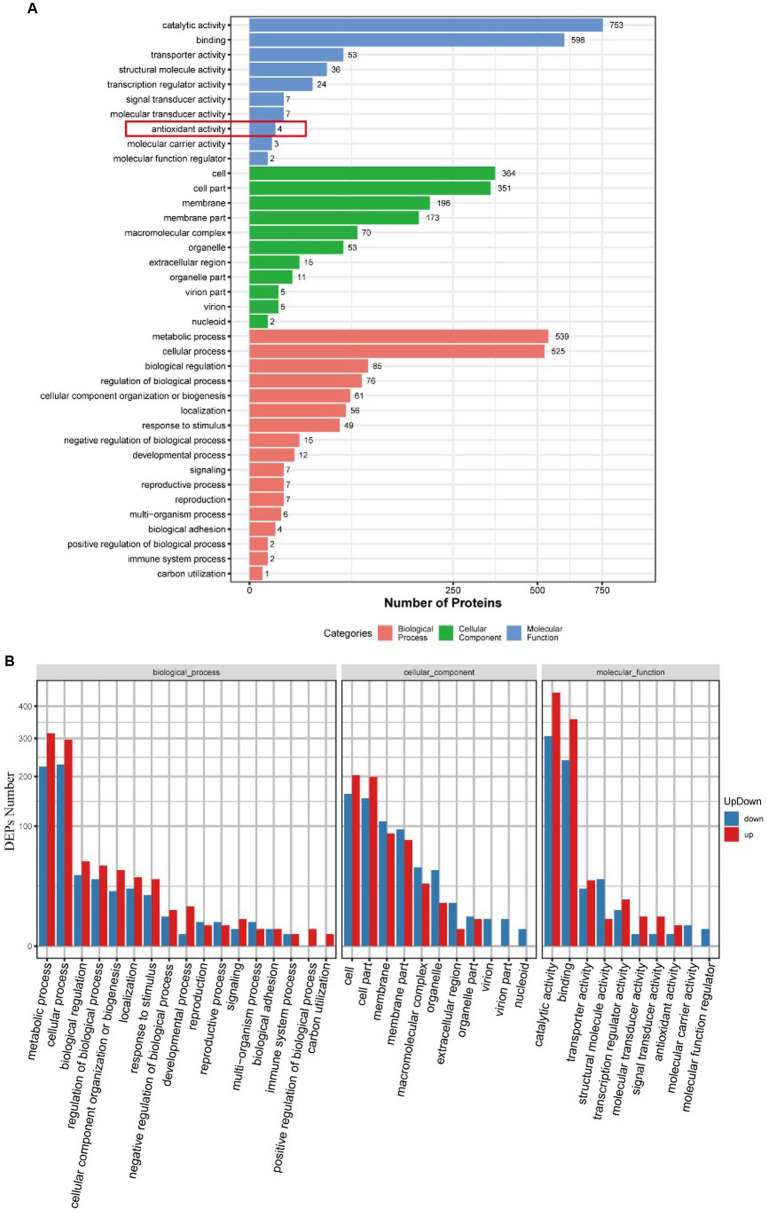
GO functional annotation and enrichment analysis of DEPs between the control and AgNPs-treated group. **(A)** GO functional annotation. **(B)** GO enrichment analysis; red: Up-regulation, blue: down-regulation.

Gene Ontology (GO) analysis classified all the identified proteins and DEPs into three broad categories: molecular function, cellular components, and biological processes (Figure 4A). Molecular functional analysis showed that AgNP treatment significantly affected catalytic activity, binding, transporter activity, structural molecule activity, and transcription regulator activity. Partial GO enrichment analysis of DEPs is shown in Table 1.

**Table 1:** GO enrichment analysis of DEPs.

Domains	Gene ontology term	Cluster frequency	Protein frequency of use	*p*-value
Cell	Cell wall	11 out of 500 proteins, 2.2%	14 out of 798 proteins, 1.8%	0.1683713
Cell	External encapsulating structure	11 out of 500 proteins, 2.2%	14 out of 798 proteins, 1.8%	0.1683713
Cell	Membrane	196 out of 500 proteins, 39.2%	308 out of 798 proteins, 38.6%	0.353027
Catalytic activity	Oxidoreductase activity	91 out of 961 proteins, 9.5%	128 out of 1,557 proteins, 8.2%	0.01349457

DEPs, differentially expressed proteins.

Figure 4B illustrates the upregulation and downregulation of differentially expressed proteins in each classification. There were 196 proteins enriched in the membrane, of which 88 were upregulated, and 108 were downregulated.

### 3.4. Proteins related to antioxidant activity

**Figure 5:**
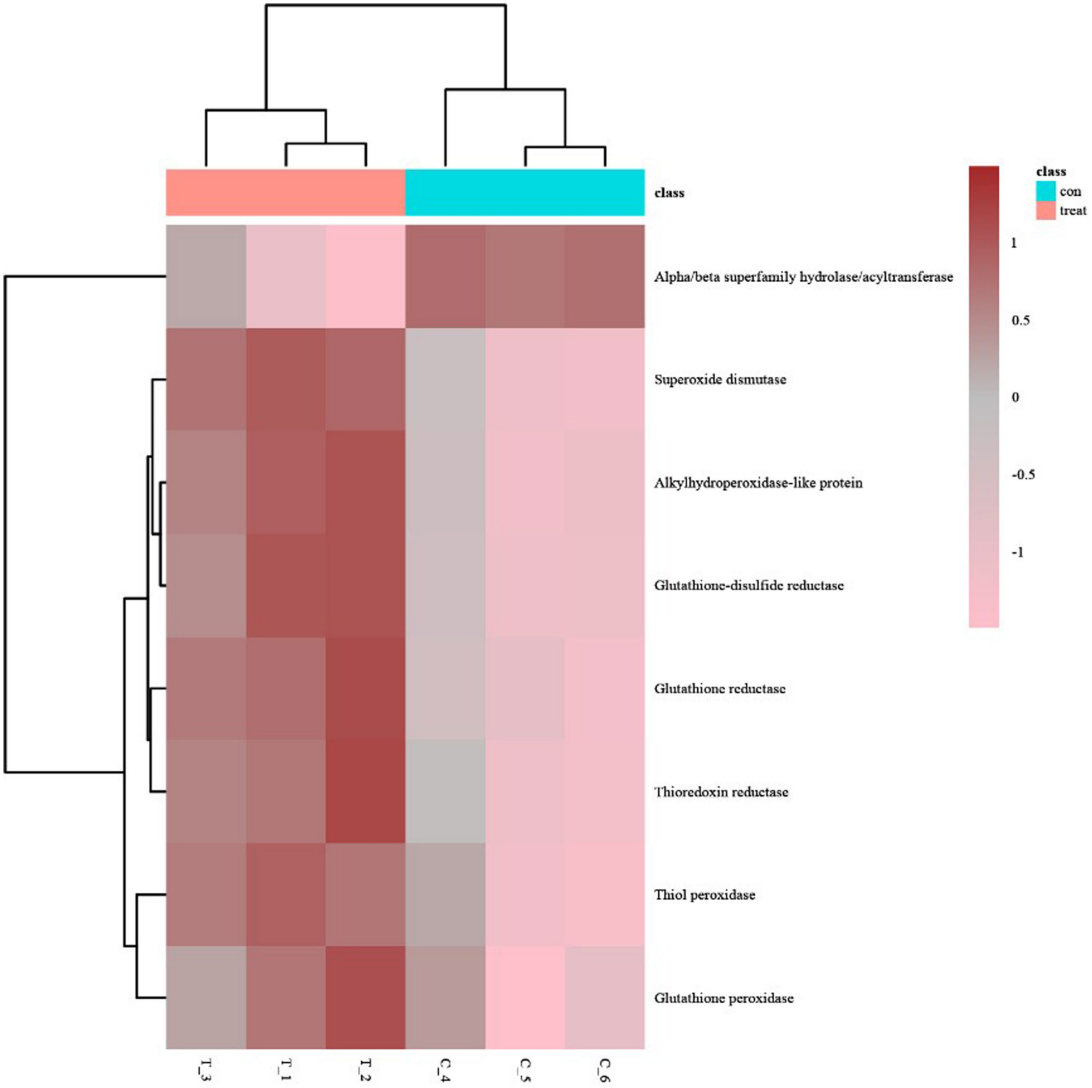
The heatmap of antioxidant activity proteins between AgNP-treated and control samples; pink indicates down-regulation, brown indicates up-regulation, and gray indicates no detectable expression change.

The heatmap of antioxidant activity proteins between the AgNPs-treated group and the control group Most of these antioxidant proteins exhibited a trend of upregulated expression, except the hydrolases/acyltransferases of the α/β superfamily (Figure 5).

GO analysis of differentially expressed proteins revealed four differentially expressed proteins enriched in antioxidant activity (Figure 4A): glutathione reductase, alkyl hydroperoxides-like protein, α/β superfamily hydrolases/acyltransferases, and glutathione disulfide reductases, of which the α/β superfamily hydrolases/acyltransferases were significantly downregulated. The remaining three proteins were significantly upregulated (Table 2).

**Table 2:** Significant differential expression of antioxidase.

Protein id	Protein name	Fold change	*p*-value
A0A0M9FKQ8	Glutathione reductase	1.251199824	0.001998308
A0A0Z8C1F7	Alkyl hydroperoxides-like protein	1.630601628	5.69E-07
A0A116KJ31	α/β superfamily hydrolases/acyltransferases	0.418632001	5.63E-06
A0A4P7WQJ0	Glutathione disulfide reductases	1.509222865	8.15E-07

### 3.5. DEPs related to cell wall and membrane

**Figure 6:**
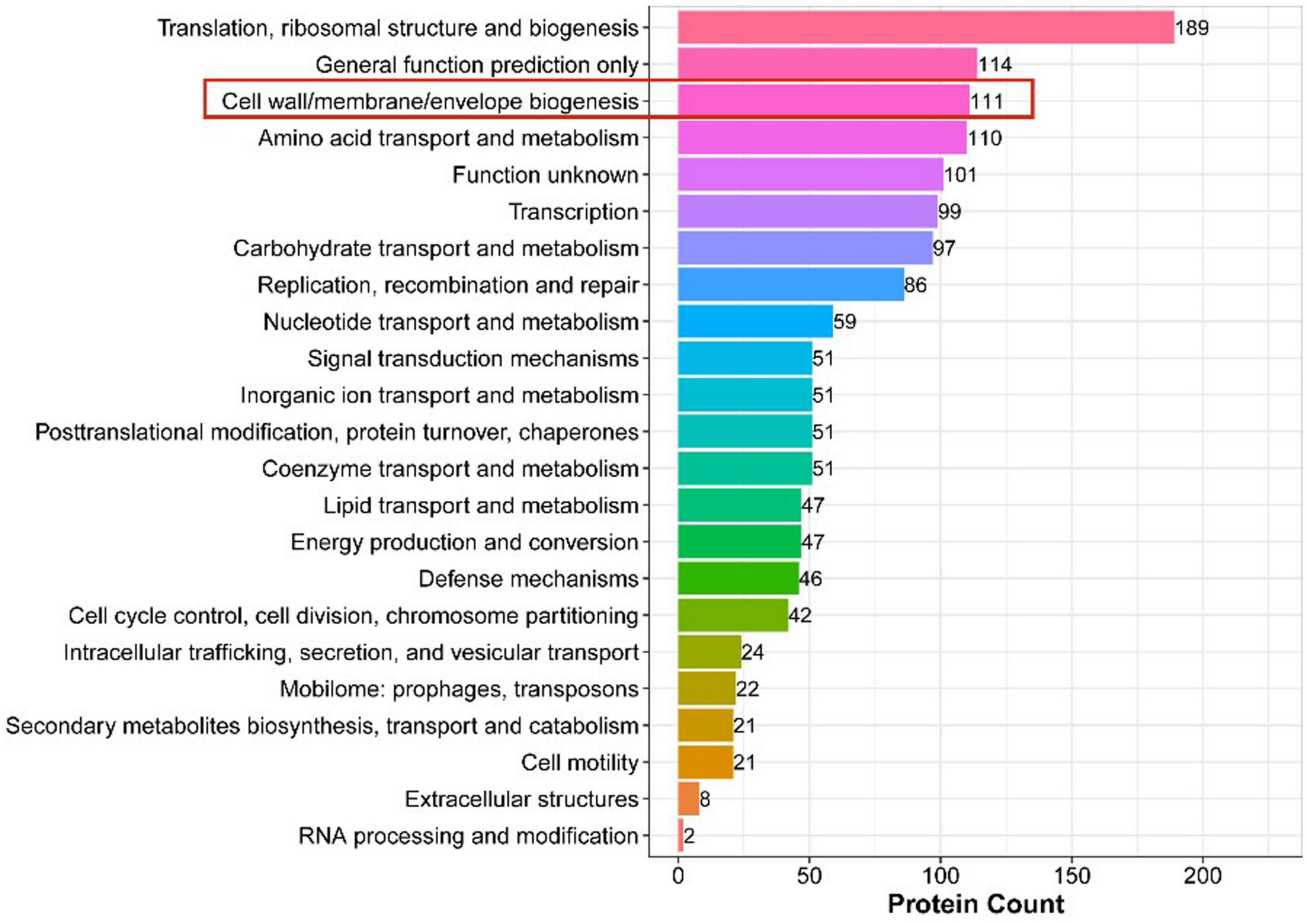
COG Annotation of DEPs. The *x*-axis displays the COG term, *y*-axis displays the corresponding protein count illustrating the protein number of different functions.

Clusters of Orthologous Groups of proteins (COGs) representing major phylogenetic lineages were delineated by comparing protein sequences encoded in complete genomes. The COG annotation of DEPs is shown in Figure 6, where among all the differentially expressed proteins, we focused on 111 proteins that were related to cell wall/membrane/environment biogenesis.

Capsular polysaccharides (CPS) are the main components of the outer capsule of the bacterial cell wall. CPS is an essential virulence factor in the pathogenesis of *S. suis* 2, and the synthesis of CPS repeating units involves multiple glycosyltransferases (Zhang et al., 2016). This analysis revealed differential expression of many CPS-related proteins (Table 3).

**Table 3:** Main DEPs that related to polysaccharide biosynthesis.

Protein id	Gene	Protein name	Fold change	*p*-value
M1VRE1	cps18L	Glycosyltransferase	3.715239563	2.13E-09
G3G7C4	CpsA	CpsA	2.694941632	3.27E-09
A0A0F6UXB6	cpsE	Polysaccharide biosynthesis protein	1.778143084	0.000124889
G8DTL5	cps1/2F	Cps1/2F	1.622947694	7.17E-08
A0A0Z8MRC6	Cps4F	Capsular polysaccharide biosynthesis protein Cps4F	1.447729138	0.000710695
A0A1C9IEG7	cpsB	Capsular polysaccharide biosynthesis protein CpsC	1.430938337	0.000679726
F8V3X0	Cps2J	Cps2J	1.378094191	2.06E-05
D5AGQ3	Cps2A	Cps2A	0.758915744	0.001492075
A0A1P8VS74	cpsM	Glycosyltransferase	0.658082439	0.000467928
M1VE61	cps34G	Glycosyltransferase	0.541453009	0.001454764
M3VAR7	cps10P	Capsular polysaccharide repeat unit transporter	0.509366561	1.75E-06
M1VK74	cps29H	Cps29H	0.374824171	1.21E-07

DEPs, differentially expressed proteins.

Peptidoglycan is an important component of the cell wall of Gram-positive bacteria, necessary for maintenance of cell morphology, size, osmotic pressure, and survival (Egan et al., 2020). Among the 111 DEPs annotated for the biosynthesis of the cell wall, cell membrane, and envelope, we found that 14 peptidoglycan synthesis-related proteins were downregulated (Table 4). Therefore, we speculated that AgNPs inhibited the synthesis of peptidoglycan, thus, affecting the normal structure of the cell wall and destroying bacterial morphology.

**Table 4:** Down-regulation of cell wall peptidoglycan-related proteins.

Protein id	Gene	Protein name	Fold change	*p*-value
A0A8D4A303	APQ97_10670	Penicillin-binding protein	0.819055107	0.000002
A0A7Y6RNH8	HU146_00905	Penicillin-binding protein	0.773657626	0.001779082
A0A3R8SHA8	EI998_07205	Penicillin-binding protein	0.25199044	0.00000239
A0A426TDI5	EI998_06570	Penicillin-binding protein	0.652981731	0.0000000366
A0A3Q8C029	A7J08_00860	Transglycosylase	0.808999066	0.0000215
A0A4V0E7P6	NCTC10237	Group 1 glycosyl transferase	0.806064227	0.0000115
A0A1P8VS74	cpsM	Glycosyltransferase	0.658082439	0.000467928
M1VE61	cps34G	Glycosyltransferase	0.541453009	0.001454764
A0A0Z8MRM4	ERS132461_01387	Glycosyl transferases group 1	0.472805889	0.00000014
D5AGQ3	SSGZ1_0555	Cps2A	0.758915744	0.001492075
A0A7G1JN94	DAT299_16280	Transcriptional regulator	0.744769672	0.00000459
A0A2I5KNA2	gtfA	UDP-N-acetylglucosamine--peptide N-acetylglucosaminyltransferase GtfA subunit	0.55881808	0.00000599
A0A4V4RY93	FAJ36_02030	LytR family transcriptional regulator	0.523049993	0.0000131
A0A123TDD7	ERS132398_01932	D-alanyl-D-alanine carboxypeptidase	0.500770473	0.000000359

### 3.6. KEGG pathway analysis of DEPs

**Figure 7:**
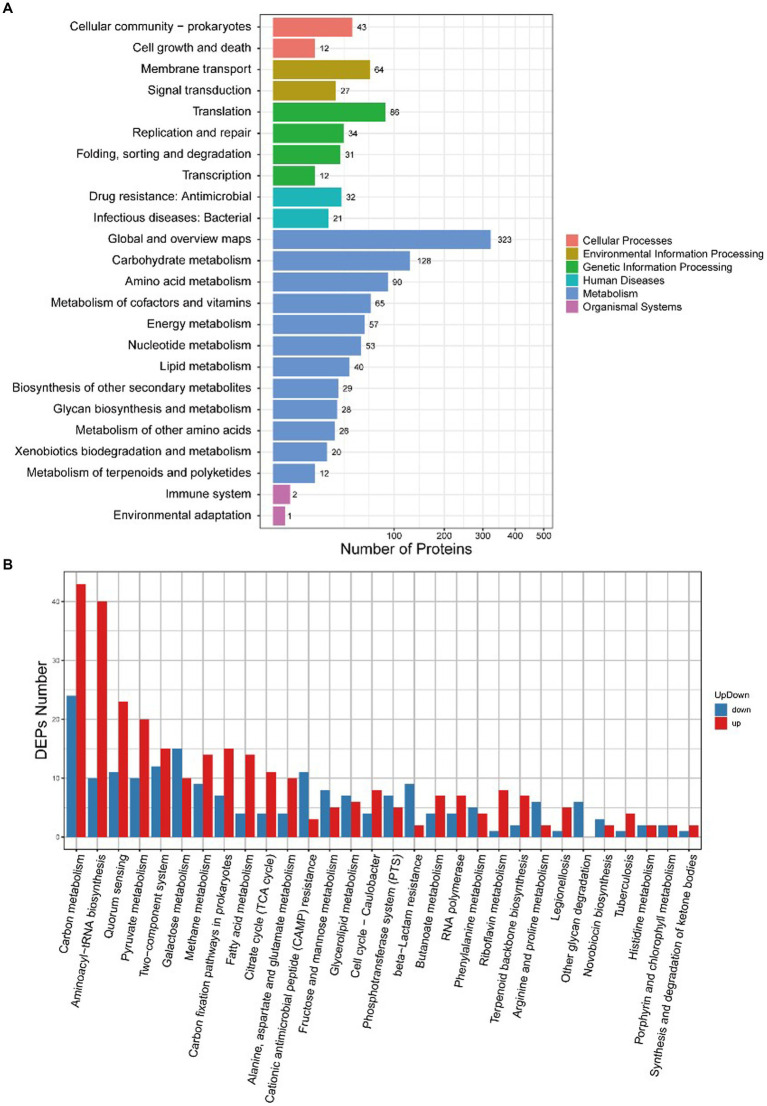
KEGG enrichment analysis of DEPs between the control group and the AgNP-treated group. **(A)** Enrichment at levels 1 and 2, **(B)** Specific enrichment pathways of DEPs.

There were 34 differentially expressed proteins enriched in the quorum sensing pathway, of which 23 were upregulated and 11 were downregulated (Figure 7). The quorum sensing system had a regulatory effect on various life activities of *S. suis*, and AgNPs interfered with the expression of related proteins in the quorum sensing system, which would affect the regulation of bacterial density of fine communities. This could potentially be one of the mechanisms through which AgNPs inhibit biofilm formation or eliminate the formed biofilms of *S. suis*.

**Figure 8:**
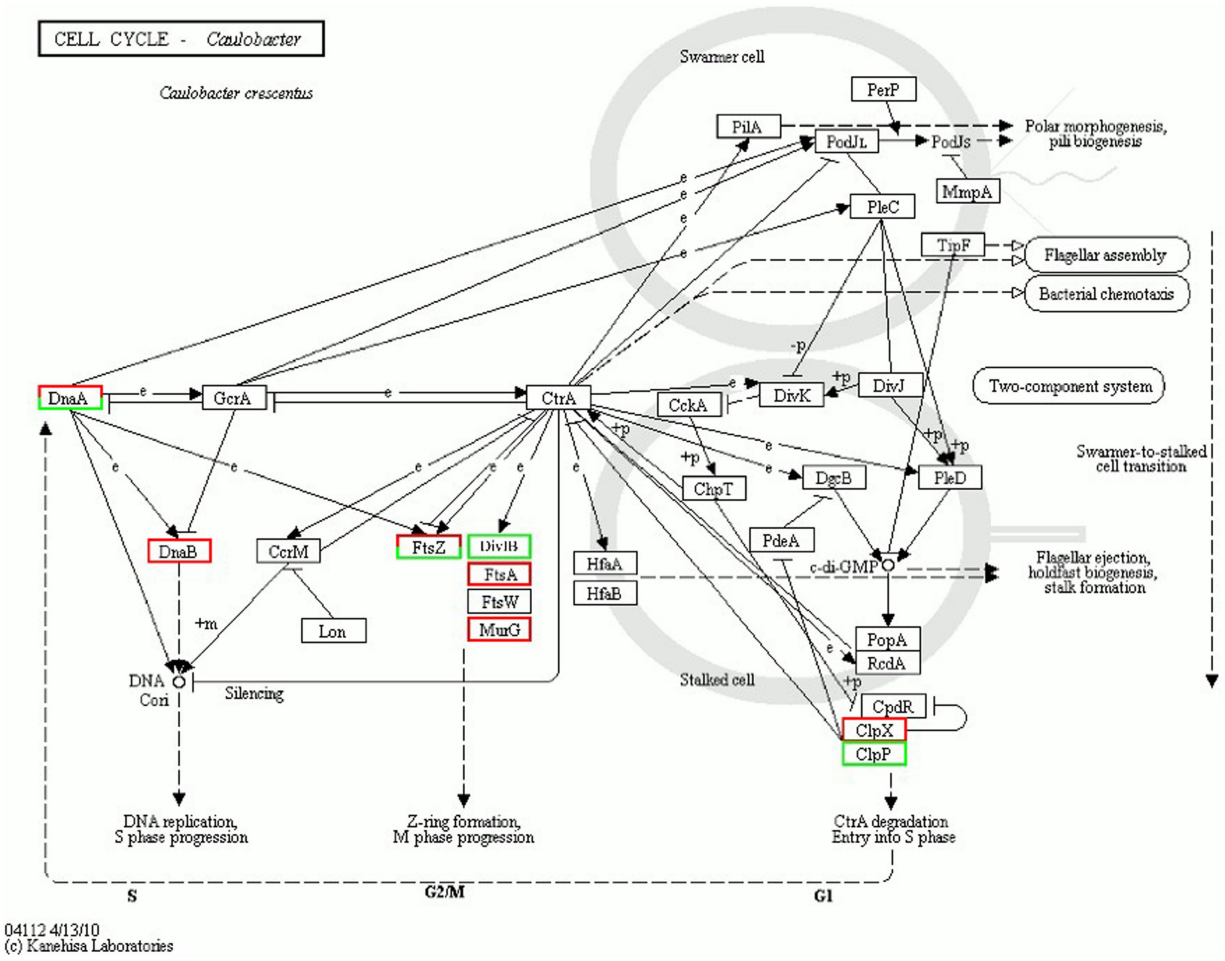
Cell cycle regulation.

DEPs enriched in the cell cycle are shown in Figure 8. Table 5 describes the significantly downregulated cell division-related proteins. This includes the chromosome replication initiator protein DnaA, cell division proteins FtsZ and DivIB, and the ATP-dependent Clp protease ATP-binding subunit. The results showed that AgNPs affected the expression of cell division protein-related proteins and inhibited the division of *S. suis* at the initial stage, which may be an important factor affecting bacterial proliferation.

**Table 5:** Significantly down-regulated cell cycle-related proteins.

Protein ID	Gene	Protein name	log2Foldchange	*p*-value
A0A4T2GNC2	ftsZ	Cell division protein FtsZ	0.77191644	0.000726526
A0A6L8MV95	DivIB	Cell division protein DivIB	0.42594536	4.86E-07
A0A4T2GJQ4	clpP	ATP-dependent Clp protease ATP-binding subunit	0.817041551	0.000199795
A0A126UJ41	DnaA	Chromosomal replication initiator protein DnaA	0.728893174	2.80E-05

### 3.7. AgNPs cause oxidative stress in *Streptococcus suis*

**Figure 9:**
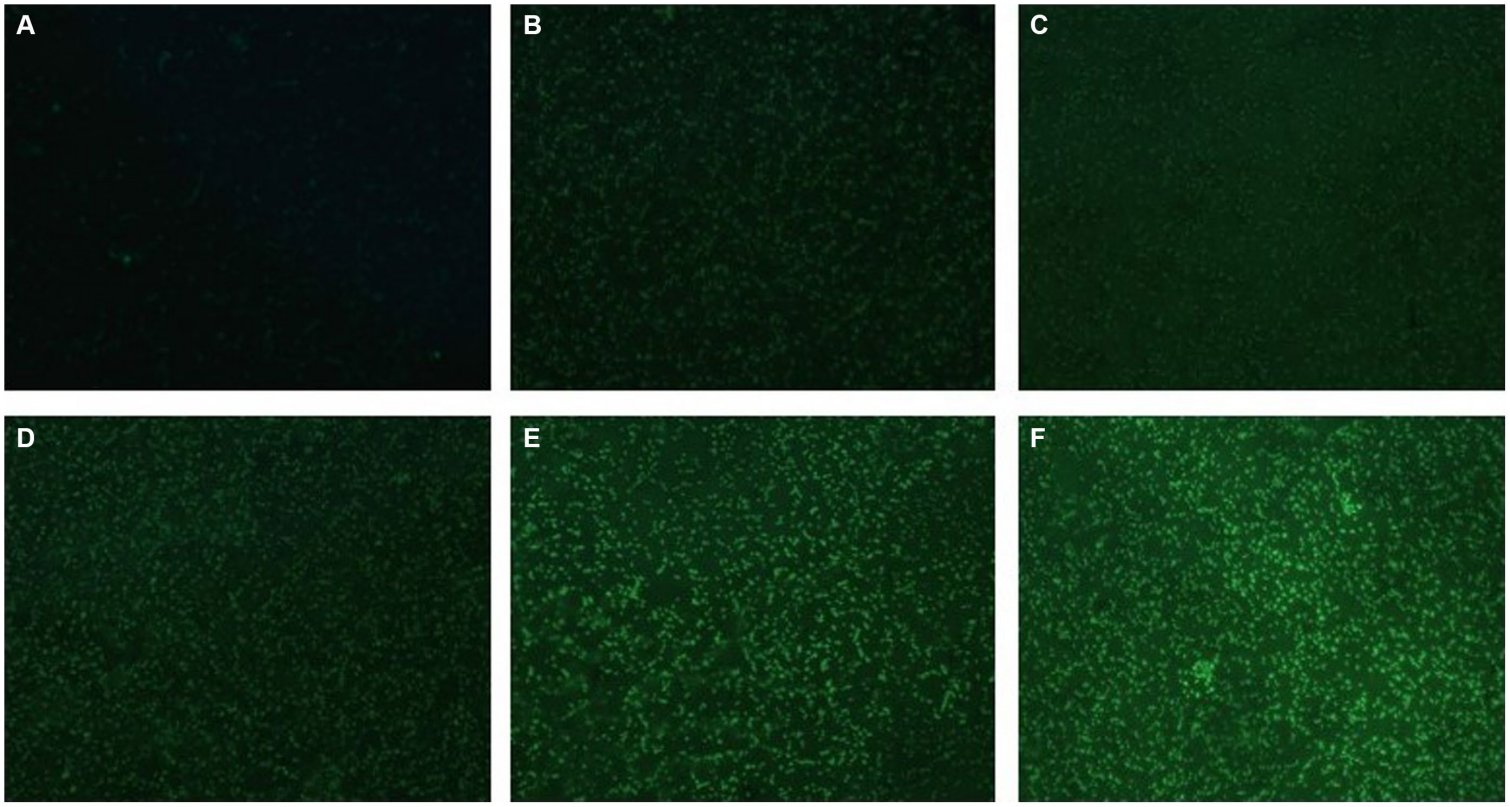
Changes in ROS production in AgNP-treated multidrug-resistant *S. suis* at different concentrations under fluorescence microscopy with ×400 magnification. **(A)** The untreated *S.suis* without observable fluorescence. **(B–F)** Fluorescence observation of the bacteria treated with AgNPs at different concentrations of 6.25, 12.5, 25, 50, and 100 μg/mL.

**Figure 10:**
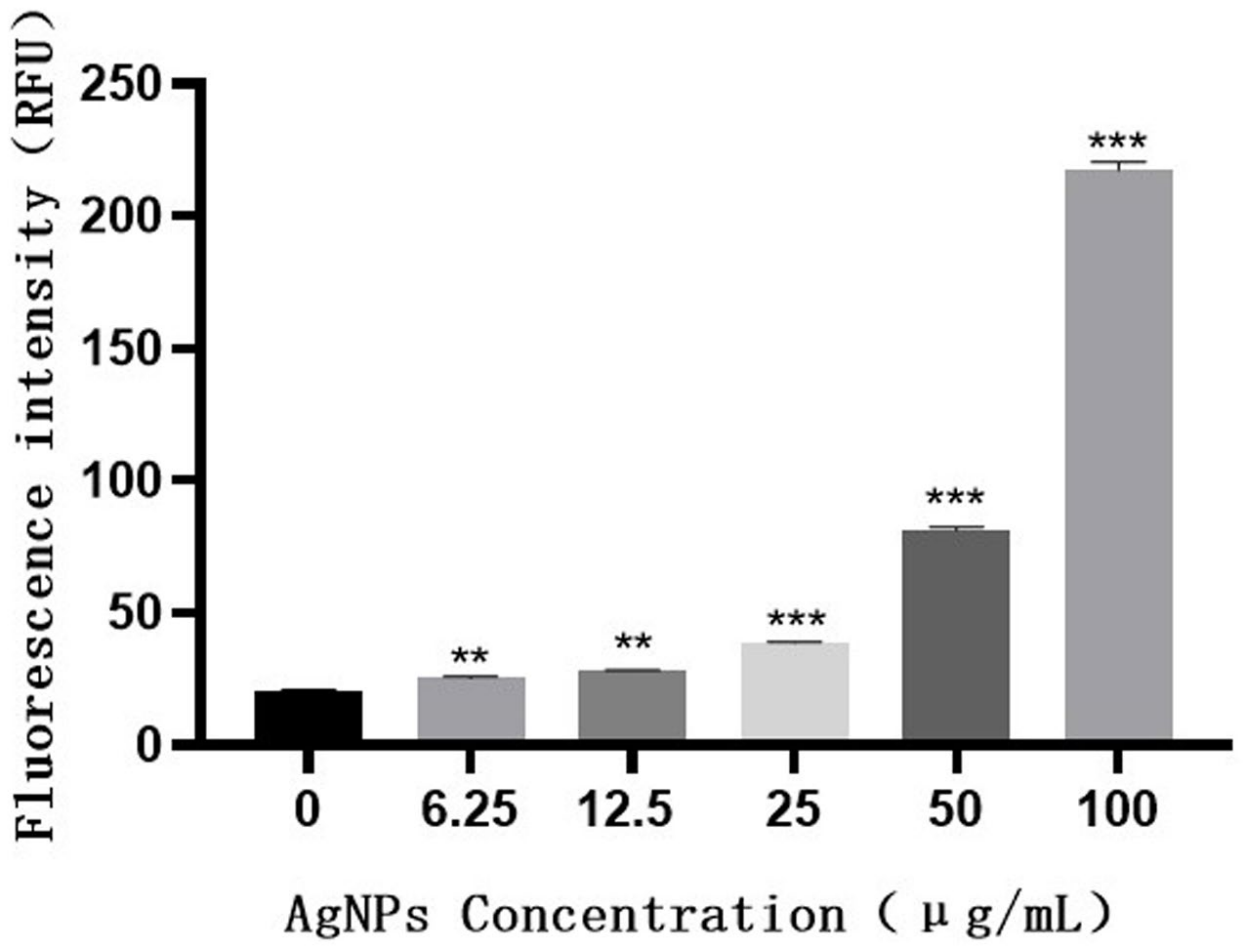
Fluorescence intensity of *S. suis* treated with different concentrations of AgNPs.

H_2_DCF-DA staining and fluorescence microscopy revealed that, compared to the weak fluorescence of untreated *S. suis*, the fluorescence intensity of the AgNP-treated bacteria increased with an increase in AgNP concentration within 6 h (Figure 9). The bacteria displayed increased fluorescence intensity when the AgNPs were added (Figure 10), indicating that the AgNPs induced ROS production in a dose-dependent manner.

## 4. Discussion

The lack of an effective vaccine to prevent *S. suis* disease has led to the widespread use of antibiotics worldwide, with the attendant problem of bacterial resistance. AgNPs exhibit antibacterial, antifungal, antiviral, anti-inflammatory, and antiangiogenic properties owing to their unique physical, chemical, and biological properties (Gurunathan et al., 2014). The antibacterial activity of AgNPs is reportedly due to the production of ROS, malondialdehyde, and leakage of proteins and sugars from bacterial cells (Yuan et al., 2017). It is well known that excessive ROS can lead to oxidative stress in cells; relevant evidence indicates that intensified oxidative stress disturbs energy metabolism and protein metabolism of bacteria (Chen et al., 2022; Zhou et al., 2023a).

In our previous study, AgNPs showed significant antibacterial and anti-biofilm effects against *S. suis* (Liu et al., 2022). In the present study, the mechanism of action of AgNPs against *S. suis* was explored based on their previously established antibacterial effects. Our results indicate that AgNPs may damage the morphology of multidrug-resistant *S. suis* and the structure of its biofilm (Figures 1, 2). The cell wall and membrane are important structures for maintenance of normal bacteria morphology. We found that many proteins related to peptidoglycan synthesis were downregulated, including penicillin-binding proteins (PBPs), glycosyltransferases, and LytR family transcriptional regulators (Table 4). The normal presence of PBPs is necessary for maintenance of normal bacteria morphology and function (Wei et al., 2003). Therefore, we inferred that the inhibition of PBP expression by AgNPs is an important factor in their antibacterial effect.

Quorum sensing (QS) and capsular polysaccharides have important influences on biofilm formation in *S.suis* biofilms. QS is a microbial cell-to-cell communication process that dynamically regulates various metabolic and physiological activities (Wu et al., 2020). LuxS/AI-2-mediated QS is a key system involved in the formation of biofilms (Wang et al., 2018). Our results identified 34 differentially expressed proteins enriched in the quorum sensing pathway, of which the expression of S-ribosylhomocysteine lyase was significantly up-regulated. S-ribosylhomocysteine lyase is regulated by LuxS, which is involved in the synthesis of autoinducer 2 (AI-2) which is secreted by bacteria and is used to communicate both the cell density and the metabolic potential of the environment. Moreover, upregulation of cps2J may promote the synthesis of capsular polysaccharides (Wang S. et al., 2017), thereby decreasing the formation of *S. suis* biofilm. These results suggest that AgNPs inhibit the formation of *S. suis* biofilms by affecting QS and capsular polysaccharide synthesis.

Cell division is an important core process in almost all organisms and is regulated by multiple genes and proteins. Bacterial cell division is coordinated by macromolecular protein complexes. DnaA is the most conserved DNA replication initiation protein, which can initiate chromosome replication and acts as a transcription factor (Menikpurage et al., 2021). FtsZ is the initiation structure of division formation and cytokinesis (Silber et al., 2020). It is an essential cell division protein that forms a contraction ring structure (Z ring) at the site of cell division. It is also an important target of antibacterial drugs (Ur et al., 2020). This study found that the expression of the chromosome replication initiation protein DnaA, cell division initiation proteins FtsZ and DivIB, and the ATP-dependent Clp protease proteolytic subunit was downregulated after AgNP treatment. These proteins are essential for bacterial replication and division; therefore, they could be important factors for the effect of AgNPs on bacterial proliferation.

ROS is an umbrella term for an array of molecular oxygen derivatives that occur as a normal attribute of aerobic life (Sies and Jones, 2020). Most living organisms possess enzymatic defenses (superoxide dismutase [SOD], glutathione peroxidase [GPx], and glutathione reductase [GR]), non-enzymatic antioxidant defenses (glutathione, thioredoxin, Vitamin C, Vitamin E), and repair systems that protect them against oxidative stress (Wang Y. et al., 2017). However, excessive ROS causes an imbalance between oxidation and antioxidation, resulting in oxidative stress that damages various cellular components (including proteins, lipids, and DNA) (Schieber and Chandel, 2014; Huang et al., 2021) and ultimately induces bacterial death. Van Acker and Coenye (2017) demonstrated that ROS mediates the bactericidal mechanisms of some antibiotics. ROS levels that exceed the capacity of the cellular antioxidant defense system induce oxidative stress (Niki, 2016). The intensified oxidative stress may also affect the structure and permeability of the cell membrane, subsequently leading to cell death (Zhou et al., 2023b).

Our results revealed that the production amount of bacterial ROS was positively correlated with the concentration of added AgNPs to some extent. Based on iTRAQ quantitative proteomic analysis, our results indicate that AgNPs significantly affected the expression of a large number of bacterial proteins, including oxidoreductases. We found that the expression of some oxidoreductases, such as GR, increased significantly, which may have been due to an increase in ROS. We speculate that the action of AgNPs leads to the enhancement of the metabolic activity of cells, which then produces excessive ROS, and the increasing ROS induces the continuous expression of antioxidant enzymes to eliminate excessive ROS. While four antioxidant enzymes were significantly differentially expressed, our results suggest that this is insufficient to effectively eliminate excessive ROS in the bacterial cells. As a result, the cells experience oxidative stress, leading to oxidative damage and, ultimately, death. These findings are consistent with previous research by Liao et al. (2019) and suggest that excessive oxidative stress is a key factor in bacterial mortality.

In summary, our findings indicate that AgNPs disturb the natural morphology of *S. suis* and its biofilm. The results obtained through proteomic analysis suggest that AgNPs may negatively impact the cell wall structure by inhibiting the synthesis of peptidoglycan, thus, leading to bacterial morphology disruption. Additionally, AgNPs can impede bacterial adhesion, interfere with QS system, and inhibit bacterial growth by targeting the cell division protein FtsZ and the chromosomal replication initiator protein DnaA. AgNPs’ induction of considerable oxidative stress is a significant contributing factor to bacterial death.

These findings contribute to a better understanding of the molecular basis of AgNPs’ antibacterial activity, highlighting potential targets for the development of new antimicrobial agents against *S. suis* infections.

## Data availability statement

The datasets for this study can be found in iProX, https://www.iprox.cn/page/project.html?id=IPX0007482000. The mass spectrometry proteomics data have been deposited to the ProteomeXchange Consortium, https://proteomecentral.proteomexchange.org, via the iProX partner repository with the dataset identifier PXD046681.

## Author contributions

BL: Writing – original draft, Writing – review & editing. DL: Writing – review & editing. TC: Writing – review & editing. XW: Writing – review & editing. HX: Writing – review & editing. GW: Writing – review & editing. RC: Writing – review & editing.
